# The Role of Knowledge Management Capability, Financial Literacy, and Problem-Solving Skills on Organizational Performance for SMEs

**DOI:** 10.3389/fpsyg.2022.930742

**Published:** 2022-10-06

**Authors:** Indro Bawono, Erna Maulina, Muhamad Rizal, Margo Purnomo

**Affiliations:** Department of Business Administration, Faculty of Political and Social Sciences, Universitas Padjadjaran, Bandung, Indonesia

**Keywords:** supply chain, organizational performance, SMEs, knowledge management capability, financial literacy, problem-solving skill

## Introduction

Small and Medium Enterprises (SMEs), as productive economic businesses, have a vital role in the economy. The role of SMEs in various countries, especially in third world countries, including Indonesia, is undoubtedly one of the supporting sectors of the economy (Eccles et al., 2014). As we know, SME business activities are very diverse with various types of goods and services produced, such as sellers of necessities, food to haircut services. In both developed and developing countries, promoting an environment that is conducive to the development of SMEs is essential. SMEs serve as the primary driver of job creation and growth in Gross Domestic Product (GDP) (Ahlin et al., 2014). They contribute significantly to economic diversification and social stability and play an important role in private sector development.

The data from Ministry of Cooperation and SMEs of the Republic of Indonesia in 2017 states that, in terms of the number of units, SMEs have a share of around 99.99% (62.9 million units) of the total business actors in Indonesia; on the other hand, large businesses only have a share of 0–0.01% or about 5,400 units. In terms of employment, micro-businesses absorb around 107.2 million workers (89.2%), small businesses 5.7 million (4.74%), and medium enterprises 3.73 million (3.11%) (Marie et al., 2014). Meanwhile, big enterprises only absorb around 3.58 million people. This may imply that, collectively, the SMEs sector absorbs around 97% of the national workforce, while large enterprises only absorb about 3% of the total national workforce. Furthermore, in terms of the scale of economic activity, collectively, the SMEs sector contributes around 60% of Indonesia's total GDP.

On the other hand, the classic problems that always overshadow SMEs are limited capital, human resources, product and technology innovation, and marketing (Kaymak and Bektas, 2017). Not all SMEs have the capital for production costs because they usually rely on private capital, which means their production costs are still limited, so they are not able to meet consumer demand optimally. Another obstacle is the relatively low quality of human resources. Other factors are the low quality of management and the lack of understanding of resource development by SMEs in facing business development constraints, including limited network expansion and the implementation of good financial management. Especially concerning capital, the government and BI have issued many policies and financing allocations for SMEs. For example, when the impact of the Coronavirus disease 2019 (COVID-19) pandemic hit the entire economy of the country, including Indonesia, the government allocated more than IDR 123T for SMEs in the framework of the National Economic Recovery Program. Yet, again, if SMEs are not ready in terms of competence and knowledge, especially in the financial sector, this has the potential to hinder the distribution of these funds.

Based on the history and survey results conducted by the OJK in 2013, the level of financial literacy in the SMEs group was only 15.68%. Furthermore, based on data from Bank Indonesia, at the end of the fourth quarter of 2018, SME credit growth grew by 9.7% when compared to the growth in the previous quarter (9.1%, yoy). However, this increase occurred in the medium business classification at 7.51% (yoy) compared to the previous quarter at 5.5% (yoy). Meanwhile, micro and small enterprises each slowed down by 12.7% (yoy) and 10.4% (yoy) compared to the previous quarter's growth of 13.6% (yoy) and 10.8% (yoy), respectively. Based on business classifications, most of the SME loans were channeled to medium-sized business loans, namely 44.5% and the rest for small businesses at 30.1% and micro enterprises at 25.4%.

Based on the results of research conducted by Bank Indonesia, it was identified that one of the constraints of banking in extending credit to SMEs is the limited banking information regarding SMEs that are potential and their eligibility, as well as low levels of financial literacy in SMEs, which have an impact on credit absorption by the banking sector. To improve this performance, it is necessary to increase the knowledge, financial literacy, and decision-making abilities of SME entrepreneurs. Hence, this study aims to develop an empirical model of strategies for improving the organizational performance of SMEs as part of improving company performance to build economic resilience in Indonesia.

## Discussion

The development of SMEs cannot be separated from their financial management problems. In running a business, of course, good financial management is needed through good knowledge and skills, which, in this case, there are not many SME players who can make it happen. The majority of SMEs are run by families with a lack of financial stability and difficulties in resolving the problems at hand. SMEs tend to lack the resources to exploit technology, resulting in low efficiency, cannot follow existing best practices, and cannot carry out further analysis to measure their performance. The need to increase knowledge for SMEs is a must to improve performance, financial performance, and overall organizational performance, create innovations, and solve problems faced.

Every business organization needs to have knowledge management capabilities to improve its learning competencies (Liao et al., 2011). Knowledge is needed to find a solution to every problem faced to be resolved immediately. Knowledge generates economic value when used to solve problems, explore new opportunities and make decisions, which will increase the company's ability to understand the environment and have experience in the future. On the other hand, the organization's time exploring knowledge for problem-solving will become a database for the company. Organizations that do not have a knowledge management system cannot develop these individuals and organizations' skills and learning abilities.

One of the knowledge capabilities that need to be improved and the most important thing related to improving SMEs' financial performance is increasing financial literacy. One of the obstacles for banks in extending credit to SMEs is the limited information from the banking sector regarding these SMEs' potential and feasibility. Increasing financial literacy will impact credit absorption by banks, considering that not all SMEs can take advantage of financial products and services. Strategies to increase financial literacy will also help SMEs in business management, starting from budgeting, planning for saving business funds, and overall financial performance. Furthermore, organizational learning ability through problem-solving activities is one of the most important strategic dynamic abilities (Purwanto et al., 2020). The learning in the future will increase the discussion in problem-solving to the level of company analysis. Successful companies learn through problem-solving either in terms of creativity in problem-solving or from the side of speed in solving them, which will make them superior to competitors (Remund, 2010).

**Figure 1:**
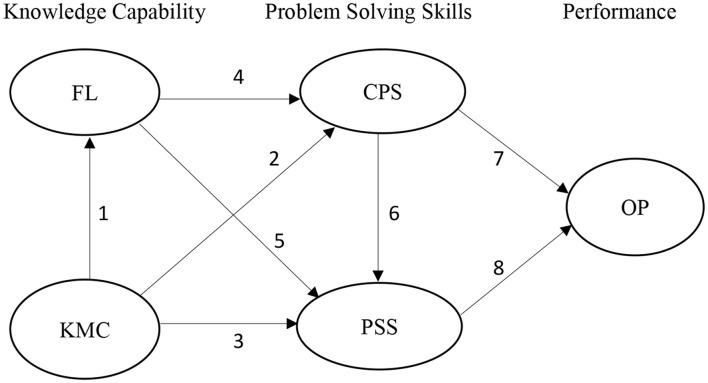
Small and medium enterprises (SME) performance improvement model. Source: Developed by author.

For this reason, success to improve the performance of SMEs requires a series of several interrelated variables and influences, starting from the knowledge management capabilities possessed, the level of literacy, which is to improve performance, is mediated by abilities both in terms of creativity, and speed in solving any problems that are encountered. This relationship can be illustrated in Figure 1.

## Conclusions

This study finds a relationship and a new working model that to improve the organizational performance of SMEs, good financial literacy and knowledge capabilities are required to be mediated by creativity and speed in problem-solving. Improving the performance of SMEs from the financial aspect is not always resolved by assisting in the form of loans, but it requires the ability of SMEs to manage and use them (Prahiawan et al., 2021).

The model confirms the impact of knowledge management capabilities on organizational performance, consisting of financial and non-financial performance. It is essential to emphasize that both knowledge and innovation are fundamental things that are useful for solving problems, and, if implemented effectively, efficiently, and sustainably, can be used as a competitive advantage. On the other hand, in case we face new problems, and beyond our knowledge, the tools we can rely on are our cognitive abilities, especially our creativity.

## Author Contributions

All authors listed have made a substantial, direct, and intellectual contribution to the work and approved it for publication.

## Conflict of Interest

The authors declare that the research was conducted in the absence of any commercial or financial relationships that could be construed as a potential conflict of interest.

## Publisher's Note

All claims expressed in this article are solely those of the authors and do not necessarily represent those of their affiliated organizations, or those of the publisher, the editors and the reviewers. Any product that may be evaluated in this article, or claim that may be made by its manufacturer, is not guaranteed or endorsed by the publisher.
